# Protocol paper for the *Movimente* school-based program: A cluster-randomized controlled trial targeting physical activity and sedentary behavior among Brazilian adolescents

**DOI:** 10.1097/MD.0000000000021233

**Published:** 2020-07-31

**Authors:** Kelly Samara Silva, Jaqueline Aragoni da Silva, Valter Cordeiro Barbosa Filho, Priscila Cristina dos Santos, Pablo Magno da Silveira, Marcus V.V. Lopes, Jo Salmon

**Affiliations:** a Department of Physical Education, Research Centre in Physical Activity and Health, Sport Centre, Federal University of Santa Catarina, Florianopolis; b Federal Institute of Education, Science and Technology of Ceara, Aracati, Brazil; c Institute for Physical Activity and Nutrition, Faculty of Health, Deakin University, Victoria, Australia.

**Keywords:** adolescent, physical activity, protocol, school-based intervention, sedentary behavior

## Abstract

**Background:**

A better understanding of how multicomponent school-based interventions work and their effects on health and education outcomes are needed. This paper described the methods of the *Movimente* Program, a school-based intervention that aims to increase physical activity (PA) and decrease sedentary behavior (SB) among Brazilian students.

**Methods:**

This is a cluster randomized controlled trial with adolescents from 7th to 9th grade in public schools from Florianopolis, Southern Brazil. After agreement, 6 schools were randomly selected to intervention or control groups (3 schools each), and all eligible students were invited to the study. The *Movimente* intervention program was performed during a school year and included 3 main components: Teacher training (including face-to-face meeting, social media platform, and handbook with lesson plans); improvements in the PA environment in school; and educational strategies. Control schools continued with their traditional schedule. Baseline (March/April 2017), postintervention (November/December 2017), and maintenance (June/July 2018) evaluations included PA and SB as primary outcomes (assessed by self-report and accelerometry). Secondary outcomes included psychosocial factors related to PA and SB (e.g., social support and self-efficacy), as well as health (e.g., quality of life and nutritional status) and education (e.g., academic achievement) outcomes. A program evaluation was performed based on the RE-AIM framework. Participants, intervention staffs, and evaluators were not blinded to group assignment, but a standardized evaluation protocol was applied independently of the trial allocation.

**Results:**

Statistical analyses will include a multilevel approach for repeated measurements and mediation analysis. Any side effects of the intervention will be recorded. The sample size close to that expected (n = 1090) was reached (n = 999). The results of this trial will involve valuable information about the effect and the evaluation of a multicomponent intervention carried out in a middle-income country.

**Conclusion:**

By creating opportunities for adolescents to be active at school using multicomponent strategies, the *Movimente* program has the potential to enhance students health and academic performance which may encourage the school community (e.g., teachers, principals) to adopt the program. Also, this trial will provide evidence for practitioners, policy makers, and researchers on how multicomponent program may be implemented in a school setting.

**Trial registration:**

The trial is registered at the Clinical Trial Registry (Trial ID: NCT02944318; date of registration: 18 October 2016).

## 1 Introduction

The investment in adolescent health is one of the priority goals of the sustainable development proposed by the United Nations in the 2030 agenda.^[1]^ In addition, World Health Organization (WHO) recognizes that increasing physical activity (PA) and reducing sedentary behavior (SB) may prevent noncommunicable diseases and morbidities in the general population.^[2]^ It has been acknowledged that promoting a healthy lifestyle to increase the population prevalence of those reaching PA recommendations^[3]^ will improve population health.^[4]^ However, to achieve a more active and healthier society, effective strategies that make PA part of daily life are necessary.^[4]^

Promoting PA and reducing SB have been strongly encouraged in low and middle-income countries (LMICs).^[5]^ Despite PA interventions in LMICs having been nominated by experts as a research question priority,^[6]^ a systematic review of PA interventions in adolescents found that only 3.8% of the included studies came from these countries.^[7]^ Multisetting interventions (e.g., family, school)^[7]^ that identify the reasons why a program succeeds or fails to encourage adolescent PA and other obesity-related behaviors are needed.^[8]^ Schools are a key setting for promoting healthy lifestyles in youth,^[9–11]^ and a bilateral relationship between health and education has been emphasized by the World Health Organization's Health Promoting Schools (HPS) framework.^[10,12]^

This proposes a healthy school environment based on 3 main dimensions: health education included in the school curriculum; improvements in the physical and/or social school environment; and actions that engage families and/or community.^[10,12]^ A recent review of interventions based on the HPS framework showed that it is effective for improving PA in students.^[12]^ Conversely, there is a need to better articulate the connection between health and education outcomes. Systematic reviews show that among 67 included studies that used a HPS framework, 56 did not indicate any educational, and/or school-related outcomes.^[10,12]^ A better understanding of health and education outcomes and how they impact each other is needed.

In addition to using effective frameworks, it is also important to investigate the mechanisms of effectiveness of interventions on PA- and SB-related variables (e.g., perceptions of self-efficacy, social support, and environment aspects), and how these variables explain changes on PA practice or SB.^[8,13]^ van Stralen et al ^[8]^ performed a systematic review on this topic and found 18 interventions had reported on mediators of PA. However, most of the potential intrapersonal (e.g., knowledge, self-efficacy), interpersonal (e.g., social support, peer, and family model), and environmental (e.g., perception and environmental characteristics) mediators associated with PA remained largely unexplored. Evidence of intervention mediators for reducing SB among young people is even more limited.^[8,14]^ Thus, identifying the complex mechanisms by which interventions achieve behavior change is important because they can identify the critical components of the program success, contributing to the improvement of new, effective, scaled-up interventions.

Based on the gaps portrayed, the objective of this paper is to provide a step-by-step description of the design of the *Movimente* Program, a school-based PA and SB intervention aimed to increase PA and decrease time spent in SB among Brazilian student's grades 7 to 9. The intervention is based on the HPS framework, focusing attention not only on the main outcomes (PA and SB), but also on potential mediating factors and health and educational outcomes (e.g., food habits, nutritional status, quality of life, and school achievement). A mixed-method process evaluation as well as maintenance of effects will also be conducted.

## 2 Methods

### 2.1 The *Movimente* Program aim and study design overview

The intervention is called *Movimente* (the Portuguese word for movement). The main objective is to promote PA, regardless of the intensity^[4]^ and reduce SB among students from grades 7 to 9 of municipal schools from Florianopolis, in southern Brazil. Additionally, the study aimed to investigate the effects of the program on the intrapersonal, interpersonal, and environmental variables related to PA and SB, and their mediating role in achieving the primary outcomes.

Complementary goals of this study were to: explore the impact of the intervention based on the HPS framework on other health outcomes (health behaviors such as eating habits, nutritional status, quality of life, etc.); examine whether changes in PA and SB may impact academic achievement (academic scores and study habits); and determine program implementation and maintenance.

The Consolidated Standards of Reporting Trials (CONSORT) recommendations were followed to guide the construction of the present study. The *Movimente* Program is a cluster randomized controlled trial, with randomization performed at the elementary school level. This study was registered with Clinical Trials (NCT02944318) and approved by the National Research Ethics System (protocol number: 1.259.910; CAAE: 49462015.0.0000.0121; date: in November 23rd, 2015). All students signed a detailed assent form and their participation was authorized by their guardians, through a consent form. Schools assigned to the control arm received the intervention and continued with their traditional schedule (their routine includes physical education—PE classes twice a week). After the intervention period is over, schools in the control group received all materials from the *Movimente* Program, and a final report informing the main results about students was delivered to the schools. Finally, we declare that the data for this research will be available on the project website: movimente.ufsc.br/ and micro data may be requested, in case of review study and metanalysis, by contacting the main author of this manuscript.

**Figure 1:**
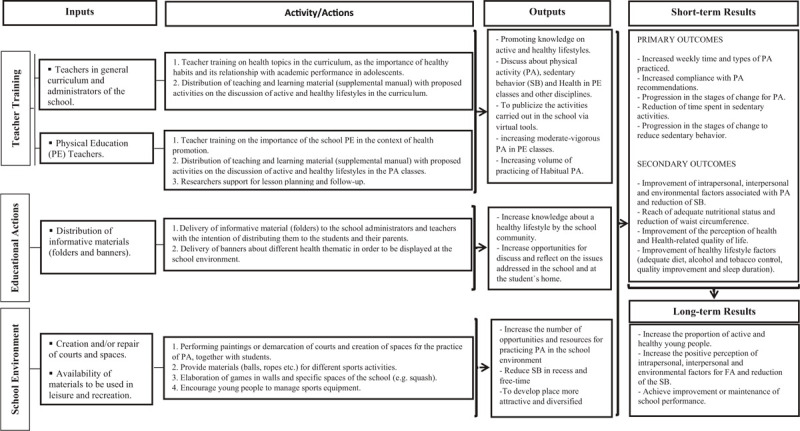
Logic model of the *Movimente* Program. PA (physical activity), SB (sedentary behavior), PE (physical education).

The program was conceptualized and designed by a research team based on previous health promotion projects in Brazil.^[15,16]^ The intervention was developed in line with the recommendations of a previous systematic review on the HPS framework.^[12]^ Guided by the *PA Evaluation Handbook*,^[17]^ the logic model of the program was developed. As recommended,^[12]^ this framework describes the program's main components as well as the interactive tools that will guide researchers throughout each step of the program (Fig. 1).

### 2.2 Power calculation

All sample size power calculations considered a statistical power of 80% and a 5% significance level for 2-tailed tests. An odds ratio of 2^[18]^ was considered meaningful, that is, students from the intervention group would be 2 times more likely to become active (self-report measure) compared with their peers from the control group. Considering these parameters and the sample design (RCT), a minimum sample of 517 subjects (1:1 between intervention and control groups) was estimated using the software OpenEpi.^[19]^ Because of the clustered nature of the sampling frame,^[18]^ the study sample was doubled to n = 1034 schoolchildren.

When estimating a sample size for PA as a continuous variable (self-report measure) using the software GPower 3.1, a sample of 1090 students has a statistical power to identify effect size equal to or higher than 0.17 (i.e., intervention vs control differences of 200 minutes per week in PA, the primary outcome) in scores in the adjusted comparisons between control (intervention and control) versus time (baseline and follow-up), considering the effect size found in a previous study using subjective measures.

Finally, considering the sample power for mediation analyses procedures (based on a simulation study from Fritz and MacKinnon ^[20]^), the needed sample to detect small mediation effects (standardized change of 0.14 or higher) when testing the mediating role of variables (i.e., social support for PA) in the intervention effect on primary outcomes is 539 for mediation analyses using PRODCLIN and 462 subjects for Bias-corrected bootstrap methods.^[20]^

### 2.3 Population and setting

Florianopolis is the capital of Santa Catarina state located in southern Brazil. It has 421,240 habitants, with a demographic density of 950 habitants/km^2^. The Medium Human Development Index (HDI) is 0.847 occupying the third position of bigger development among all Brazilian municipalities; with GINI Index of 0.54 (range: 0 to 1—where zero represents the situation of total equality of income). The proportion of children aged 11 to 13 attending the final grades of secondary school is 93%.^[21]^ In 2015, students in the final grades of secondary education scored 4.6 (the highest score is 5) in the Basic Education Development Index (IDEB), ranked in the 1630th position of 5570th among Brazilian municipalities.^[22]^ In 2017, 50,404 secondary school enrollments were registered in 124 schools.^[23]^ According to the Brazilian National School-based Health Survey^[24]^ 61% of the adolescents from the 9th grade did not engage in 60 minutes daily moderate-to-vigorous PA (MVPA), and 49% spent 2 hours or more in screen time per day.

A total of 36 secondary schools are under the jurisdiction of the Municipal Secretary of Education, Florianopolis.^[25]^ In this study, inclusion criteria for schools to be in the study included: having secondary level grades (27 schools out of 36); schools that had, at least,2 classes from 7 to 9 grades (to meet sample size required) (21 schools remaining); schools that were not under environment reform/repair (18 schools remaining). Thus, 18 schools were considered eligible.

### 2.4 Recruitment of schools and participants

The study was approved by the Municipal Secretary of Education, Florianopolis and recruitment of schools occurred from October to November 2015. An invitation was sent to the 18 schools by email, asking the principal of each school if they were interested in being part of the *Movimente*, irrespective of the study condition (intervention or control). Out of 18 schools, 7 agreed to participate in the research. One school (n = 6 classes) was selected for conducting the pilot study, and 6 schools were matched (number of classes and geographic location) and randomly assigned to intervention or control arms. This matched process was adopted to avoid contamination between groups (intervention and control) and guarantee an equal distribution of schools from different geographic areas in both the groups. Also, we matched schools considering the sample size (based on the number of classes) because it varied greatly between schools (from 7 to 13 classes). The socioeconomic backgrounds and teaching schedules of these schools are the same. They were all municipal public schools, with a target audience that is very similar in terms of economic and human development index. Thus, the allocations were made to get a peer-grouped (1:1 ratio) aimed to achieve a balance between trial levels (3 schools in intervention group and 3 in control group), considering the number of classes in the schools (2 schools had between 6 and 8 classes; and 4 schools had between 9 and 11 classes).

To avoid contamination between control and intervention groups, schools were not informed about which group they were in until after baseline data collection. All students in grades 7 to 9 from the 6 selected schools who attended the first weeks of school (1427 students) were eligible to be part of the program (intervention = 796 and control group = 631). In baseline data collection, a total of 370 parents did not deliver their consent after 5 attempts to send the consent form for their children and a telephone call attempt. Fifty-eight students declined to be part of the research, and 999 adolescents (response rate = 70%) completed the baseline questionnaire (intervention group n = 580; response rate = 72.9%; control group: n = 419; response rate: 66.4%). Adolescents with mental and/or physical disabilities as well as those who did not attend the first 3 weeks of the school year (period of the collecting data) were not eligible.

### 2.5 Theoretical basis of the *Movimente* program

Several program frameworks and theories of behavior change informed development of the intervention. The HPS framework^[12]^ informed the overall intervention based on 4 main dimensions: inclusion of health education topics into the school curriculum; provision of health opportunities at school through social and/or physical environment; family engagement; and communities and/or other components that may be related to the students’ behavior.^[12]^ Socioecological frameworks propose that behavior is influenced at multiple levels; and these levels interact to influence an individual's behavior: intrapersonal (e.g., attitude, self-efficacy); interpersonal (e.g., social support, modeling); and environmental factors (e.g., school equipment and facilities).^[26,27]^ Two theories of behavior change were considered. Social cognitive theory considers that behavior is influenced by both intrinsic and extrinsic factors,^[28]^ and the Transtheoretical Model takes into account several nonlinear stages when an individual aims to change their behavior.^[29]^

### 2.6 Intervention protocol

#### 2.6.1 Pilot phase

A pilot study was conducted from March to July in 2016 to prepare for the larger-scale program. During this phase, the measurement protocols were developed and tested. A total of 251 students from grades 7 to 9 were included in the sample. The research team assessed the feasibility of conducting all measures in the school (e.g., time taken to complete the questionnaire, unexpected events, etc.). Based on the pilot phase, several refinements were made, for instance: accelerometry data collection (further information to students to increase compliance); administration of the questionnaire (increasing the time allocation for completion); changes on the content of the teacher's handbook (based on general classroom teachers’ and PE teachers’ feedback).

#### 2.6.2 Intervention

The *Movimente* Program was conducted over 1 school year (March–December 2017). It is a multicomponent program comprised of 3 main components: teacher training; environmental improvements; and education curriculum (Table 1). All materials are available on the website (http://movimente.ufsc.br).

**Table 1:**
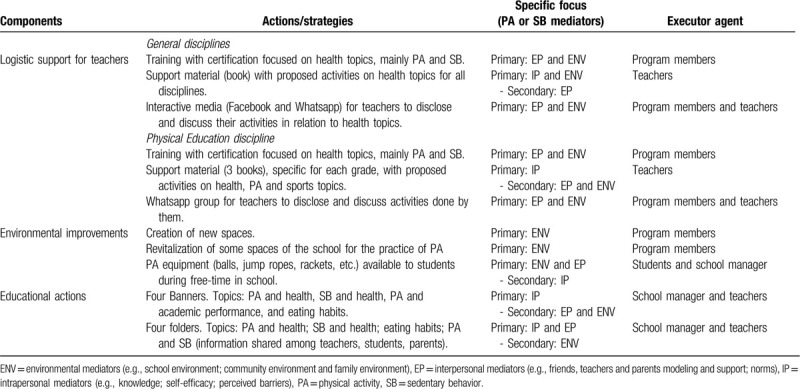
Description of the components, strategies, focused physical activity, and sedentary behavior mediators, and executor agent of the *Movimente* program (Florianopolis, Brazil, 2017).

#### 2.6.3 Teacher training component

The objective of providing teacher training was to inform and/or refresh the main goals of the program, to present several adolescent health topics and an explanation about the handbook. Teachers were encouraged to talk about health with their students, by improving their necessary skills to become the providers of change on the student's behavior. Teacher training was developed and conducted separately for general classroom teachers (e.g., Math, Portuguese, Arts), and for PE teachers. This was conducted separately as it would be expected that PE teachers already have a deeper understanding of the health impacts of PA and SB, due to their specialization with these subjects.

#### 2.6.4 General teachers

Classroom teachers from grades 7 to 9 from the intervention schools were invited to take part in the training session, which was organized in 3 stages. The first session was a 4-hour face-to-face meeting. To reach these teachers, different training days were offered. Those who agreed were enrolled in a session at school to discuss health (e.g., definition, relevance, among other topics) and its influence on all aspects of students’ life such as their behavior during classes, concentration, study habits, and academic performance.

A supplementary handbook was provided aimed at assisting teachers with several lessons plans regarding health concepts. The material was presented to teachers and its content was discussed to demonstrate several possibilities of activities to be conducted in the classroom, in different subjects. This material was adapted from a previous work,^[15]^ elaborated by the research team during monthly meetings over a year (2014/2015), taking into account either the National Curriculum Standards and the cultural aspects from Florianopolis, Santa Catarina. The handbook was divided into several sections: introduction; purpose and methodology; breaks activities guide; and 1 chapter for each of the school disciplines (e.g., Maths, Portuguese). Thus, sessions were provided by 8 activities suitable for each discipline, which were structured as follows: purpose, required material, development and suggestions to adapt the activity, and complementary text.

The session regarding breaks activities guide consisted of concepts, aims, and examples of: relaxation and stretching breaks; muscle activation breaks; and energizers breaks. Teachers were informed that the material was flexible. In other words, they could adapt the lesson content to suit the needs and abilities of their class.

Logistic support was also provided to teachers via an online social media platform and mobile phone applications aimed at stimulating dialogue between teachers from all schools and researchers. Teachers were asked to report on activities they implemented over the year and to share their experiences. This virtual platform allowed teachers to support each other and assist if they had questions relating to how to conduct certain activities. Furthermore, if required, teachers were provided ongoing support by the research team, both in person or by telephone. All activities performed by the teachers were based on a predetermined workload. At the end, the activities performed were summed, to receive the training certificate. The last stage was a face-to-face 2-hour meeting with individual interviews with each teacher. Teachers discussed the barriers, facilitators, and intentions to continuing using the activities in their school routine. Teachers who completed at least 75% of the training received a certification from the Federal University of Santa Catarina.

#### 2.6.5 PE teachers

Training for PE teachers was focused on implementation of activities and how to use the content in their classes. Activities were focused on how to engage a broader number of students in PE classes and providing activities that increase students’ enjoyment of PE classes. PE teachers were also trained on how to discuss intrapersonal aspects (e.g., attitude, self-efficacy, enjoyment) that reinforce the relevance of including PA practice in their routine as well as how to overcome barriers, and include topics on health into PE classes. The training session had the same structure as the general classroom teachers as well as a face-to-face 4-hour meeting. There was only 1 or 2 PE teachers per school, so it was decided to approach them together to allow a deepen discussion. The meeting was held at the university campus (an average distance for all of them).

PE handbooks were created and distributed to all PE teachers. They had 3 versions, each one tailored to each grade school year (7th, 8th, and 9th grades) and was composed of 4 units (Unit 1: Physical Activity; Unit 2: Life and Health; Unit 3: Sports; Unit: 4 Body and Rhythmic Practices). This material was developed based on a previous work developed in Fortaleza, Brazil,^[15]^ other educational documents such as National Curriculum Standards and Common National Curricular Basis,^[30,31]^ and specific PE literature^[32]^ was consulted. Each unit was composed of 8 chapters, organized by supporting text and suggestion of activities (aim, required materials and activity description, and suggestions for adapting the activity).

#### 2.6.6 Environment component

At each school, empty places were filled with more than 1 set of line markings (e.g., volleyball, squash, and popular games). Painting revitalization was also made in the already existing courts. A kit of sports equipment (rackets, jumps rope, balls to play basket, soccer, volley, etc.) was provided in each school. In this case, the principal decided the best way to manage this kit was for students’ use before- and after-school class and also during recess breaks (in Brazil, students have 15 minutes of recess after three 45-minute regular classes followed of more 2 regular classes). To raise student's awareness about the environmental changes and provision of sports equipment, banners were placed at each school reporting on the available materials. PE teachers were also encouraged to use the new line markings and sports equipment during their classes to disseminate and empower the students on how to use it.

#### 2.6.7 Health education component

The research team developed informative folders and banners composed of health messages, based on previous material from the *Fortaleça sua Saúde* program.^[15]^ The development phase involved a multidisciplinary team, and a preliminary version was analyzed by the teachers from the pilot school to verify its suitability. There were 4 thematic folders for parents: information about the importance of PA practice and encouraging parents to motivate and practice with their child; information about the relationship between SB and unhealthy outcomes; encouraging parents to seek alternative activities to avoid prolonged sitting time; tips on how to prepare healthy meals; messages to avoid processed food and a brief explanation on the difference between natural and processed foods; and additional messages encouraging parents to help their child to participate in more PA and avoid SB. Every 2 months, teachers received folders to be worked in the classroom, and then hand over the folders to the students, which in turn should be delivered to their parents.

There were also 4 thematic banners: information about benefits from being physically active and an encouraging message “Let's move for a healthy life;” benefits from reducing sitting time; the positive and negative aspects of a healthy and unhealthy dietary intake, respectively; and the relationship between PA and study habits and academic scores. At the beginning of the intervention, the 4 banners were delivered to school principals. They were advised to select a place with good visibility of the banners.

### 2.7 Data collection

#### 2.7.1 Training session

The staff team was composed by master (n = 5) and PhD (n = 3) students in the PA and health field. Most have been involved in data collection involving school-based intervention. In the pilot phase, all staff members attended training sessions to become familiar with the Program assessment protocols through practice sessions, calibration, and/or standardization of the measures (weight, height, and waist circumference), and of the application of the questionnaire. They received an instruction material containing a step-by-step to be followed throughout the collecting data. This material contained information about how to explain to the students the correct way to fill out questionnaires and how to save/organize the questionnaires at the end of the application session. All aspects were discussed point by point to guarantee the standardization of the procedures.

#### 2.7.2 Procedures and measures

**Figure 2:**
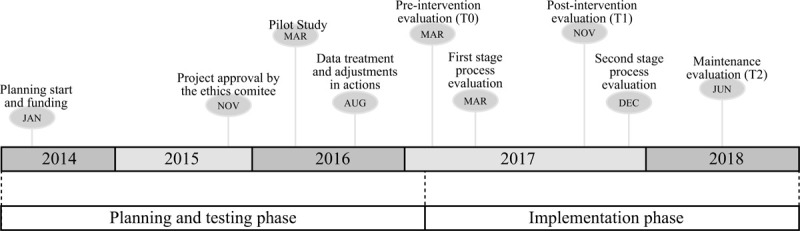
Description of the timeline of *Movimente* Program.

Figure 2 includes information regarding the timeline of the phases of this research. Assessment has been conducted with all students during 3 times points: T0 baseline preintervention (March/April 2017); T1 postintervention (November/December 2017); and T2 maintenance evaluation (June/July 2018). The T1 assessment provided an effectiveness measurement of the program and the T2 assessment provided information regarding the sustainability of the program over time even in the absence of the research team. In the first stage (March) of the process evaluation, the planning of the actions with the whole school community was investigated, and in the second moment (December), the implementation of the program was evaluated.

During the baseline, a self-completed student questionnaire was administered in class during school hours, over a mean time of 90 minutes. Consistent instructions were provided by a researcher who facilitated survey completion with further information about each question. Two other researchers also helped students that had particular questions. Additionally, it was sent a questionnaire to be filled in by parents about their children's health-related quality of life (KIDSCREEN 27). In the postintervention and maintenance phases, the same questionnaires were reapplied. Besides that, before, during and at the end of the intervention, questionnaires were applied to evaluate the intervention.

Measures reliability of the adolescent's questionnaire was evaluated (Table 2). Cronbach's alpha was applied to analyze the internal consistency of latent scales in the baseline sample. The reproducibility of measures was evaluated in the pilot sample by using Cohen's Kappa and the Gwet Concordance Coefficient on nominal variables. Weighted parameters were used on ordinal variables. The Gwet parameter was included due to known limitations of Kappa on evaluating reliability of 2×2 tables,^[33]^ providing a less biased parameter.^[34]^ Intraclass correlation coefficient and Spearman correlation coefficient were applied on continuous variables. The Spearman parameter was applied due to highly skewed distributions observed in some measures (e.g., volume physical activity).

**Table 2:**
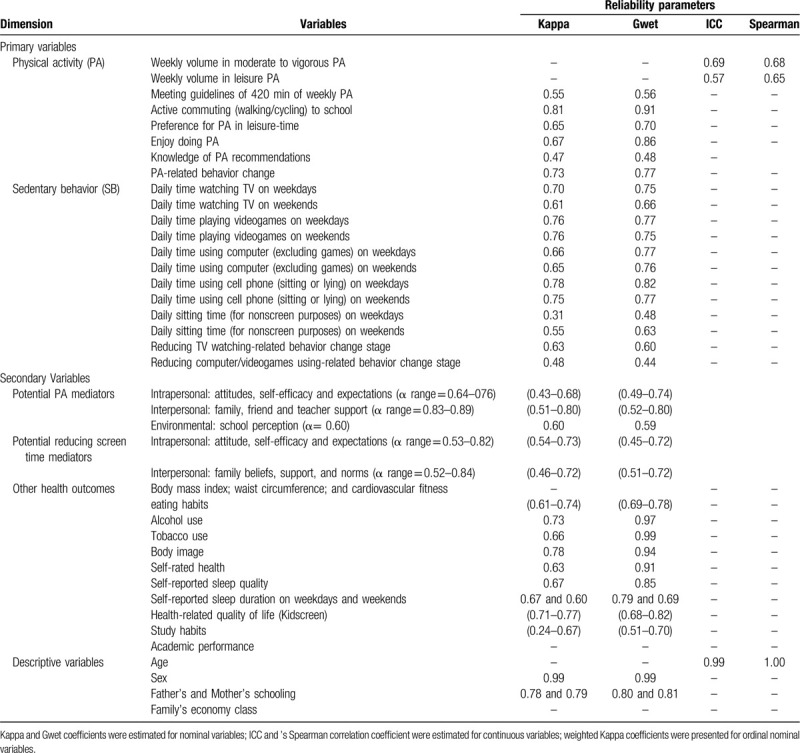
Measured variables in the *Movimente* program (Florianopolis, Brazil, 2017).

#### 2.7.3 Student questionnaire

##### 2.7.3.1 Sociodemographic characteristics

Students reported their sex, and their date of birth was obtained from the school. Socioeconomic levels were collected via an instrument made available by the Brazilian Association of Research Companies.^[35]^ Students reported the education levels of their mother and father (never studied; completed or incomplete: elementary school, middle school, university; don’t know). They also completed a 12-item list about the amount of goods (e.g., TV, car, electronic tablets, freezer, etc.) in their home. This instrument generates a general score based on reported amount of assets.^[35]^

### 2.8 Primary outcomes

#### 2.8.1 Physical activity

Students reported participation in a previously validated list of 22 different types of PA (with an option to add extra activities). Students reported whether they usually participate in any of the listed activities in a typical week, the weekly frequency and daily duration considering 2 different criteria: 1 list regarding leisure-time and another list considering total-time PA.^[15,36,37]^ This instrument showed high levels of reproducibility and a moderate level of reliability for PA dichotomous measurement. Validity of the tool against 24 hours recall was moderate and agreement in the 2 categories at 300 min/wk was moderate.^[36]^

Due to a limited number of accelerometer devices, a subsample was selected to objectively assess PA. In each group (intervention and control), schools with the smallest number of students were used as the inclusion criteria. Thus, 315 students were considered eligible. Of these, 156 control students were enrolled in a school from Southern region, while 159 intervention students were enrolled in a school located in Northern region. A total of 250 students (intervention = 136, control = 114) wore an accelerometer (response rate of 86.8%) at baseline. A detailed explanation was provided to students within class time. Participants were asked to wear ActiGraph accelerometers (GT3X+ and wGT3X+ ^[38]^) which have been widely used in large-scale studies to assess PA^[39]^ and validated for the pediatric population.^[40]^ Adolescents were instructed to wear the accelerometer on a belt positioned on the right hip over a 10-day period and only remove them for water-based activities and to sleep. Students who did not provide enough data (≥ 3 weekdays and 1 weekend day for at least 8 hours a day) were asked to rewear the device for 10 more days. The data were collected at 80 Hz and analyzed in epochs of 15 seconds, as recommended for the pediatric population.^[40]^ Periods of ≥60 minutes of zero values were defined as accelerometer nonwear time. Thresholds of movement counts (corresponding to moderate- and vigorous-intensity physical activity) in 15 second epochs developed by Evenson et al,^[41]^ an accurate threshold for this age group, will be used. From the data analysis, the main outcome will be average daily volume of PA (light PA and MVPA). Data from the accelerometers was transferred to the software Actilife 6.8 to perform reduction and validation procedures.^[42]^

#### 2.8.2 Sedentary behavior

Daily hours in SB on typical weekdays and weekend days were assessed with the following items: television viewing; virtual games (at computer and/or video-games); computer use (excluding games activities); and cell phone use (when it was the sole activity being performed while seated or lying down, i.e., cell phone use while walking was not included). Moreover, participants self-reported their overall hours of SB (e.g., sitting talking with friends, playing cards, etc.), considering the sum of all items. This instrument showed significant correlation coefficients for total screen time use. Objectively assessed sedentary time was collected based on the accelerometer data. Sedentary time will be defined as < 100 counts per minute.^[41]^

### 2.9 Secondary outcomes

#### 2.9.1 Intrapersonal, interpersonal, and environmental correlates of physical activity

Physical activity correlates were assessed through an instrument developed by Farias Júnior et al^[43]^ for adolescents aged 14 to 19 years old. These consisted of outcome expectations, self-efficacy, perceived neighborhood environment, and social support from parents and friends. This instrument has acceptable validity and reproducibility for a younger Brazilian population (12–15 years).^[44]^ The questionnaire comprised 9 items with 4-point Likert scales as follows: outcome expectations (10 items), attitude (5 items), self-efficacy (8 items), and social support from parents (6 items) and friends (5 items). Three additional items were also included: social support from PE teachers (5 items) and general teachers (5 items), and perception of the school environment (4 items).

Questions about knowledge and enjoyment regarding PA as well as leisure-time PA preferences were also included.^[37]^ Stages of behavior change regarding PA,^[29]^ mode, frequency, and duration of commuting to school^[15,18,37]^ were also asked.

#### 2.9.2 Intrapersonal, interpersonal, and environmental correlates of screen time

Adapted from the questionnaire of the “*Fortaleça sua Saúde*” Program, the present instrument was comprised of 40 items structured in 7 scales. An expectation scale assessed students’ perception of the positive (8 items) and negative (4 items) expectations about their screen time use. The attitude scale included 3 items regarding aspects for reducing screen time use, considering contrasting adjectives: fun–boring, important–negligible, health–harmful. Other scales had the response options in a 4-point Likert scale. The self-efficacy scale for reducing screen time use included 11 items and evaluated the perceptions of the students about confidence and abilities to reduce their screen time use. Students’ perceptions of family rules of screen time use (6 items), and the family's beliefs of screen time use (3 items) and support for reducing screen time (5 items) were also assessed. Stages of behavior change aimed at reducing computer use and television viewing time (less than 2 h/d) were assessed. Most of the questions were obtained from Brazilian standardized questionnaires.^[15,37]^

Validity and internal consistency were evaluated, and the results showed that the instrument had acceptable validity and reliability for Brazilian students. Exploratory factor analysis indicated that the scales were adequate according to Kaiser Meyer-Olkin index and Bartlett Sphericity Test (higher than 0.60) and Bartlett sphericity test (*P* < .001). In addition, 8 of 9 scales had adequate Cronbach's α values ranging from 0.70 to 0.85. The intraclass coefficient correlation of the scales ranged from 0.63 to 0.78 for reproducibility.

### 2.10 Health and educational outcomes based on the HPS framework

#### 2.10.1 Academic performance

Study habits^[45]^ and grades were obtained to determine academic performance.^[46]^ A Study Habits Scale composed of 6 questions with a 5-point scale (completely false to completely true) explored students’ perceptions about their study habits.^[47]^ This instrument was translated and validated (Cronbach alpha = 0.74 and composite reliability = 0.70) for Brazilian adolescents.^[45]^ Additionally, mathematics and Portuguese language grades (ranging from 0 to 10 points) throughout of the year were requested from the Department of Municipal Education and/or provided by the Director of each school. The school- and grade-specific z-scores in Mathematics and Language will be used as academic performance variables.

#### 2.10.2 Health-related quality of life (HRQoL)

The HRQoL self-report instrument (KIDSCREEN-27) for children and adolescents (from 8 and 18 years old) is a European measure with translation in several different languages, including Portuguese.^[48]^ A proxy measure to parents and caregivers also is available. The 27-item version was used in the current study, which covers the following dimensions: physical well-being, psychological well-being, autonomy and parent relation, peers and social support, and school environment. Students responded to questions about their PA and health (n = 5), general mood and feelings about themselves (n = 7), family and free time (n = 7), friends (n = 4), and school and learning (n = 4). Parents/caregivers also responded to these questions about their child, recalling the last week (i.e., how your child is, how does she/he fell, etc.).^[49]^

A Brazilian study demonstrated that KIDSCREEN-27 presented acceptable psychometric indicators of reproducibility (intraclass coefficient correlation = 0.70, 95% CI: 0.70–0.96), internal consistency (composite reliability index = 0.90, varied from 0.65 to 0.70 in the domains), and construct validity (confirmatory factor analysis = Factor loads were greater than 0.40, except for 5 items (0.28–0.39), and the model's goodness-of-fit indices were adequate) for adolescents from 10 to 15 years of age.^[50]^ In baseline, the KIDSCREEN-27 was completed by 921 adolescents (response rate of 92.2%).

#### 2.10.3 Anthropometry

Adolescents’ body weight (kg), height (cm) (both used to calculate body mass index), and waist circumference (cm) were measured in a private room, by 45 minutes. Students were asked to take off shoes and wear light clothing. Body weight was measured once using a calibrated scale to the nearest 0.1 kg. A portable stadiometer (Altura exata brand) was used to measure height to the nearest 0.1 cm. Weight status was calculated based on the body mass index (BMI = kg/m^2^) by sex and age, based on the WHO cut-off points (http://www.who.int/growthref/en/, accessed on October 02, 2017). Waist measurement was taken at the narrowest point between the inferior rib border and the iliac crest, using an inelastic tape measure. Three measures of the circumference were recorded to the nearest 0.1 cm (if the discrepancy between the first 2 were lower than 1%, it was considered the average, otherwise, the median value of all the 3 measures was observed). All protocols were guided on international standardizations.^[51]^ Anthropometric measures were completed as follows: weight (n = 859; 86%); height (n = 861; 86%); and waist circumference (n = 861; 86%).

#### 2.10.4 Physical fitness

Cardiorespiratory fitness (CRF) was assessed by the Progressive Aerobic Cardiovascular Endurance Run (PACER), a submaximal test adapted from the test of 20-m shuttle run.^[52]^ This test was validated for Brazilian adolescents, it is considered the best field test to measure CRF in adolescents.^[53,54]^

The test consists of 20-m shuttle run with a beep that indicates when the runner should reach or touch the demarcated ends. The initial speed is 8.5 km/h with a gradual increase of 0.5 km/h every minute. The test ends when the participant cannot reach the end twice before the beep. Different indicators will be analyzed as laps stages, minutes, and maximum oxygen consumption (VO_2_max) estimated by the Leger et al equation.^[52]^ The cardiorespiratory fitness test was administered to 779 students (intervention = 475 and control = 304) (response rate of 78%).

#### 2.10.5 Other lifestyle variables

Self-reported frequency of consumption of fruit, vegetables, savory snacks, sweets, and sugar sweetened beverages in a typical week was assessed.^[15,24,37]^ The consumption of alcohol and tobacco on the last 30 days was also included.^[15,37]^ Sleep quality and duration on both weekdays and weekend days were self-reported.^[15,37]^ Body image was reported by self-assessment of 9 different silhouettes, each represented by a number (from 1—thinner to 9—fatter). The following questions were asked: which image is the best representation …of your physical appearance?... of a healthy body? …of the body you would like to have?.^[55]^

### 2.11 Process evaluation

Protocol compliance (dose delivered and fidelity to each component) was determined during the intervention period using quantitative and qualitative methods. The quantitative evaluation was conducted by a previously validated instrument created for the *Movimente* Program, based on an evaluative matrix, taking into account the RE-AIM dimensions.^[56]^ The evaluation was conducted by the research team with data collected from students (during classroom time), teachers (at school), and parents (by telephone), over 3 stages, as described below.

#### 2.11.1 Planning evaluation

A questionnaire was administered at baseline which aimed to explore the parents’ and teachers’ sociodemographic features; their interest in engaging in the program as well as perceived barriers and motivations to be part of the program. *Process evaluation:* A questionnaire was administered postintervention which aimed to explore the perception regarding the importance and quality of the strategies, their engagement (teachers only) as well as perceived barriers and facilitators (teachers and parents only), and, finally, their intentions to continue being part of the program (teachers only).

Interviews were also conducted with teachers and coordinators to gain a deeper understanding of their perceptions of the program. They were interviewed one-on-one and perceptions, opinions, and barriers to implementation of the program were discussed. The interviews were conducted using a semistructured interview-guide during school time, lasting approximately 15 minutes. Conversations were recorded and consent was given after transcription.

### 2.12 Data treatment and statistical analyses

The tabulation of questionnaire data will be performed using an optical reading using the software “SPHYNX.”^[57]^ At the end of the process, the tabulation will be manually checked by 2 staff members of the Movimente study team. The management and processing of the database as well as the descriptive analyses will be performed in the statistical package “STATA Standard Edition 15.”^[58]^ Inferential analyses will be performed in both STATA and R Project for Statistical Computing 3.5.1 software.^[59]^

Descriptive analyses will be performed to describe the sample, presenting mean values for continuous variables and absolute and relative frequency for qualitative variables, both with their respective 95% confidence intervals (95% CI). Pearson *χ*^2^ tests will be used to compare sociodemographic characteristics among adolescents with valid PA accelerometer data to those without valid data.

The multilevel approach for repeated measurements will be applied through the application of generalized linear mixed models in the inferential analyses of PA and SB (primary outcomes). The models will be tested in relation to their assumptions of normality and independence of residues, as well as checked in relation to homoscedasticity. If these assumptions are not met, linear models for different families (e.g., Gamma) will be tested that are better suited to the features of the modeled variables. Transformation of non-normally distributed variables will be performed where appropriate. In addition, Akaike information criterion (AIC), and Bayesian information criterion (BIC) will be applied to evaluate models fit.

Regarding mediation, the following steps will be followed: action theory test (Pathway A: to investigate the impact of the intervention on hypothesized mediators at time 2, controlled for baseline values); conceptual theory test (Pathway B: to analyze the association between mediators and the dependent variable at time 2, controlled for group assignment and baseline values); to verify the total effect (Pathway C), direct effect (Pathway C’: analyze the effect of the intervention that is not explained by the mediators) and indirect effect (Pathway AB: the mediated effect) of the intervention for each outcome; a significance test of the mediated effect and the proportion mediated [AB/ (C’ +AB)] were calculated. Then, a multiple mediator model will be performed by including the significant mediators.^[60]^

## 3 Discussion

This paper provides an overview of the *Movimente* Program, describing the step-by-step development of a multicomponent PA and SB intervention. There are few theoretically based PA and SB interventions in schools.^[9]^ Moreover, the *Movimente* Program is based on a whole-school approach, inspired by the HPS framework, which considers education beyond the classroom, expanding its actions to the social and physical school environment.^[10,12]^

Multicomponent interventions (i.e., actions for the school community and parents) are generally more effective at changing behavior than simple interventions.^[7]^ However, evidence is needed to identify which strategies are effective in changing these PA and SB. Another consideration in developing intervention strategies is whether the actions will have external validity; that is, whether they can be delivered in the real world. The *Movimente* Program focused on external validity given it is being conducted in a real-world setting. The program was designed to be implemented by teachers and school principals.

In this study, the intervention strategies covered 3 important pillars: educational strategies; teacher training; and environmental changes. A systematic review has shown positive results when strategies are combined and directed to intervene both to increase PA and to reduce SB in adolescents.^[9]^ However, few intervention studies have targeted both PA and SB,^[61,62]^ particularly in low and middle-income countries (LMIC). For instance, one review of PA interventions observed that only 3% of 75 included studies were conducted in LMIC, with only 2 from Brazil. All 16 studies that targeted SB were conducted in high-income countries (6 studies in European countries, 1 Australia, 9 in the United States).^[63]^ Brazilian data were not explored in previous reviews,^[64,65]^ but 3 intervention studies targeting children's SB in Brazil have been identified.^[66–68]^ Therefore, a greater number of intervention studies in LMIC like Brazil are required to ensure evidence of intervention effectiveness in those countries.

Incorporation of potential mediators in the present study is important for understanding the mechanisms of change and what factors directly or indirectly influenced a specific outcome. For instance, according to previous evidence of their role in interventions^[9]^ students’ attitudes, enjoyment, and social support may be extremely important for PA participation. Data on this topic will enhance our understanding about psychosocial mechanisms of behavior change,^[69]^ and will contribute to improving the development of future interventions in this field.^[70]^ However, research on mediators of SB is very limited, with very few instruments to evaluate mediators of this behavior. Therefore, the present study sought to explore this issue as well.

The *Movimente* Program also seeks to target health- and education-related outcomes, like indicators of mental health, physical and social health, and aspects of health-related quality of life. This comprehensive focus is justified by the need to understand the individual as a complex being who receives influence and is influenced by the environment in which he or she lives.^[4]^ Such information will provide a whole view of how the intervention might impact adolescent health, beyond the target outcomes.^[9]^ Another priority of the *Movimente* Program is to observe if, in some ways, health aspects can impact education^[71,72]^ as highlighted in the Sustainable Development Agenda from United Nation Development Program.^[73]^

Studies have shown that PA is positively associated with academic performance.^[74,75]^ Reading fluency (grades 1–3) and arithmetic skills (grade 1) were also inversely associated with sitting time in boys.^[76]^ Intervention studies have identified significant beneficial effects of physical education classes^[77]^ and PA^[78]^ on academic performance in adolescents. Finally, it is also necessary to investigate types of discretionary (e.g., sitting while watching television, playing video games, etc.) and nondiscretionary (e.g., sitting during work or school) SB^[32]^ and their impact on school performance.

Another important aspect of the intervention is evaluation of the program implementation as well as effectiveness. The mixed-methods provide a comprehensive understanding of implementation,^[79]^ including both quantitative and qualitative evaluation.^[9]^ Process evaluation will be performed with quantitative and qualitative measures to verify the feasibility of the program and the perceptions of participants with different levels of involvement (school principals, parents, teachers, and students). Additionally, the long-term evaluation is another strength, since it will help to explore the sustainability of the program.^[9]^

Limitations include the self-reported primary measures that might result in memory bias or social desirability. However, the instruments were previously validated for Brazilian young people.^[44]^ Another concern is that health initiatives occurred in both intervention and control schools during the program and also addressed health topics. Brazilian government supports 2 national-wide health programs: the “More education program,” which was suspended in 2017 in the city of *Florianópolis*, and the “School Health Program.” However, both programs occurred in all included schools in a similar way and, thus, it may not influence in the analysis of the intervention effect. Instead, we believe further intervention in Brazil should consider a closer partnership with nationally health program (e.g., “School Health Program”), because they may improve a favorable environment for physical activity and health promotion. Finally, cost-effectiveness was not evaluated.

By creating opportunities for adolescents to be active at school through games, improving PE classes, having active breaks and learning about health in class, the *Movimente* Program has the potential to enhance students’ health and academic performance which may encourage the school community (e.g., teachers, principals) to adopt the program. Future results will also enhance understanding of PA and SB mediators and will provide evidence for practitioners, policy makers, and researchers of the effectiveness of this multicomponent school-based program.

## Acknowledgments

The authors thank the Municipal Education Department of Florianopolis for the technical support and authorization to execute the Program. The authors thank all members of the school community (managers, teachers, parents, and students) of the schools involved by the support during the execution of the program; the Sports Center of the Federal University of Santa Catarina for technical support. The authors also thank the *Movimente* Program working group: Management Team: Kelly S. Silva, Adriano F. Borgatto, Maria A. A. de Assis, Valter C. Barbosa Filho, Jaqueline A. Silva, Priscila C. Santos, Pablo M. Silveira. Executing Team: Alexsandra S. Bandeira, Marcus V. V. Lopes, Bruno G. G. da Costa, Soraya A. Sá, Rafael M. da Costa, Margarethe Knebel, Gabrielli T. Mello, Luís E. A. Malheiros, and Thiago S. Matias. All persons mentioned have allowed their name to be included in this section.

## Author contributions

**Conceptualization:** Kelly Samara Silva.

**Formal analysis:** Marcus Vinicius Veber Lopes.

**Methodology:** Kelly Samara Silva, Jaqueline Aragoni Silva, Valter Cordeiro Barbosa Filho, Jo Salmon.

**Supervision:** Jo Salmon.

**Validation:** Jaqueline Aragoni Silva, Valter Cordeiro Barbosa Filho, Priscila Cristina dos Santos, Pablo Magno da Silveira, Marcus Vinicius Veber Lopes.

**Writing – original draft:** Kelly Samara Silva, Jaqueline Aragoni Silva, Valter Cordeiro Barbosa Filho, Priscila Cristina dos Santos, Pablo Magno da Silveira, Marcus Vinicius Veber Lopes, Jo Salmon.

**Writing – review & editing:** Kelly Samara Silva, Jaqueline Aragoni Silva, Valter Cordeiro Barbosa Filho, Priscila Cristina dos Santos, Pablo Magno da Silveira, Marcus Vinicius Veber Lopes, Jo Salmon.
